# The past and present of pandemic management: health diplomacy, international epidemiological surveillance, and COVID-19

**DOI:** 10.1007/s40656-021-00416-4

**Published:** 2021-04-30

**Authors:** Flavio D’Abramo

**Affiliations:** Max Planck Institute for the History of Science, Berlin, Germany

**Keywords:** COVID-19, History of epidemiology, Health diplomacy, Epidemiological surveillance, Colonialism, International sanitary conferences

## Abstract

The establishment of international sanitary institutions, which took place in the context of rivalry among the great European powers and their colonial expansion in Asia, allowed for the development of administrative systems of international epidemiological surveillance as a response to the cholera epidemics at the end of the nineteenth century. In this note, I reflect on how a historical analysis of the inception of international epidemiological surveillance and pandemic management helps us to understand what is happening in the COVID-19 pandemic today.

International relations play a central role in the scientific coordination necessary to halt the contagion of COVID-19 across the world. The two-week delay by Chinese authorities in notifying the World Health Organization of the outbreak of an uncommon form of pneumonia was the first element of international contention that emerged at the beginning of 2020. The US government leveraged that delay to claim compensation from the Chinese government, with the argument that an earlier notification of the outbreak would have stopped the spread of the virus and spared the death of hundreds of thousands of Americans (Yang 2021). In this short piece, I build upon my previous scholarship on empirical medical ethics and the history of biomedicine to reflect on the origins of the first international epidemiological surveillance system, ideated during the International Sanitary Conferences between 1851 and 1938. In doing so, I provide a much-needed historicization of the US-China contention over sanitation, and show the deceptive quality of political powers’ appeals to the “non-political” to doing something “humanitarian” and “scientific” precisely where their economic self-interest is involved (D’Abramo et al. 2020). Namely, national rivalries and international power imbalances have characterized European epidemiological surveillance from its beginning.

Historically, international trade and epidemics went hand in hand. Indeed, since the foundation of the maritime republics of Venice, Pisa, and Genoa, commercial fleets of European powers reaching far-away countries proved to be a harmful vector of contagion (Harrison 2013; Marchini 2004; Watts 1999). Quarantine, which from the fourteenth century onwards began to be one of the most widespread means to halt contagion, was also a means of war that came along with the making of European nation states.^1^ The international management of health emerged only later, in the nineteenth century. When the hundreds of thousands of losses caused by cholera epidemics and the improper use of quarantine converged with the 1831 Egyptian invasion of the Ottoman province of Syria, which posed a direct threat to British and French commerce, the European powers turned to diplomacy (Harrison 2013). In 1851, diplomats and scientists of the European powers, Turkey, and Russia met in Paris at the first International Sanitary Conference organized by France and Great Britain, aiming to coordinate international trade in the Mediterranean Sea. There, it was declared that “politics and all that relates to it must be carefully and expressly excluded from the deliberation” (Conférences Sanitaire Internationale—Ministère des Affaires Étrangères 1851, p. 5—Septième Séance). In those times, the evidence of microbes as factors determining ill health was yet to be discovered. Furthermore, the use of quarantine was neither further adopted nor ratified, both to protect the commercial and economic activities that would have been affected by it as well as to shield against the possibility that quarantine would be used arbitrarily.^2^

At the 1892 Venice International Sanitary Conference, the scientific evidence developed a few years before by Louis Pasteur and Robert Koch about biological agents causing contagious diseases gained ground. Delegates convened in Venice to discuss the quarantine as a part of the activities of the Egyptian *Conseil Sanitaire Maritime et Quarantenaire*, an international institution meant to regulate the traffic of the Suez Canal, through which commercial and military vessels of France, Britain, and other European powers reached Asian countries. The use of bacteriological analysis, steam sterilization, and medical inspections were conceived as ways to alleviate the use of quarantine and to safeguard the flow of commerce. In 1891, physician Justin Karlinski reported on the disastrous conditions of the Egyptian medical station of El-Tor, on the Red Sea, where Bosnian and Herzegovinian pilgrims were quarantined on their way back from Mecca. In the drinking water of the camp’s containers Karlinski counted more than “2 million germs per cubic centimetre” (Conférences Sanitaire Internationale 1892, p. 89). By appealing to the achievements of “men of science,” the Italian delegate Count Francesco Antonio d’Arco limited the aim of the conference “to determining the practical application of the data acquired by a system that would have the least possible interference with the freedom of communication and commerce” (Conférences Sanitaire Internationale 1892, p. 5). D’Arco called for a humanitarian, philanthropic ethos underpinned by bacteriology and microbiology: “From the shores of the Adriatic, European civilisation has for centuries flown towards Oriental regions which today call for our attention and which will be the subject of our studies. But this time it will not be the flag of St Mark that they will see arriving: It will be the flag of a great humanitarian idea, borne by the coalition of States that are marching at the head of progress” (Conférences Sanitaire Internationale 1892, p. 6). The Austro-Hungarian delegate Franz von Kuefstein added that the sanitary danger was indeed represented by the “growing development of relations with the Extreme Orient […] and needed a humanitarian, unitary mission against a common enemy that escaped any isolated action” (Conférences Sanitaire Internationale 1892, p. 7). The French delegate Professor Adrien Proust expressed concerns about the vessels in transit in the canal due to frequent contact between the crews and locals, and called for the establishment of a “serious system of surveillance in the Suez Canal.” To mitigate the British rationale, which frequently subordinated quarantine to trade and thus contributed to worsening the health of many people, Austro-Hungarian diplomats agreed with British politicians in 1891 to establish a system of telegraphic surveillance. That system was eventually adopted at the 1892 Venice conference. The agreement was meant to help maritime powers represented at the Egyptian Sanitary Council to monitor infected or suspected vessels so as to avoid any contact with Mediterranean ports not included in the routes stated by the captain at the Council’s maritime station, in Suez. Each government was empowered to enact discretionary penalties against vessels which, abandoning the course declared at the Council, unduly approached one of the ports in the territory of that country.

In 1893, at the International Sanitary Conference of Dresden, diplomats and physicians adopted the first international epidemiological surveillance system to prevent the spread of cholera epidemics in Europe. Indeed, between 1817 and 1893, five cholera outbreaks hit Asia, Africa, Europe, and the Americas, bringing their medical, social, and economic systems to their knees. The delegates who convened at the conference, Robert Koch included, unanimously decreed that telegraphic notification among diplomatic and national offices was the most important international protective measure against cholera. The compulsory weekly notification included the existence of a cholera outbreak on land, the precise place where it occurred, its starting date, the number of clinically observed cases, and the number of deaths (Conférences Sanitaire Internationale 1893).

The epidemiological surveillance system of 1893 protected Europe from the sanitary crises of its colonies, and contributed substantially to the misattribution of responsibility for cholera epidemics to social groups that suffered from the disease without having any power to halt or slow the contagion. Despite the fact that John Snow revealed cholera’s means of diffusion in 1849, and that Filippo Pacini discovered its biological agent in 1854, the colonial administrators of Great Britain refused to recognize the structural, environmental causes of cholera outbreaks (D’Abramo et al. 2020). Namely, instead of acknowledging vibrio cholera leaking from the sewers and open fields into the aqueducts and irrigation canals of India, colonial governors misattributed the spread of cholera to the allegedly unhygienic habits of the Indian people. In doing so, they avoided tackling the material and logistic aspects of the gigantic structural reform of aqueducts and irrigation canals necessary to stop the epidemic (Watts 1999). Still, as late as 1862, the treaty-port city^3^ of Tianjin in China, where seven European powers had obtained economic and political concessions in order to take advantage of the burgeoning markets of salt, textiles, and grain, was hit by the Cholera epidemic that had started in India and had later moved along the network formed by the British empire (Rogaski 2004).

The European powers’ rejection of critical self-reflection in the nineteenth century produced a legacy of misguided assumptions about Western medical and administrative superiority whose consequences extend to the current health crisis. Looking at what has unfolded in the course of the COVID-19 global health crises since late 2019, one could argue that the same kind of perceived hegemony still animates many of today’s escalating disputes between the US and China. Over the last hundred years, politicians and diplomats, supported by administrative and biomedical experts, have contributed to the development of epidemiological surveillance systems in order to trace, isolate, cure, and prevent cases of infections and epidemics (D'Abramo and Neumeyer 2020; D’Abramo et al. 2021—forthcoming). Yet, it seems that political rivalries and the resulting lack of international coordination and cooperation remain mostly unsolved, even today. While a worn-out “epidemic Orientalism” leads European and American politicians to blame China and Asian countries for having generated the pandemic and for denying civil rights through severe lockdowns (Rudyak et al. 2021), the self-image of liberal, enlightened societies needs to be amended, and the history of health diplomacy is a convenient vantage point to do so (Brazelton 2020; de Almeida 2015; Vargha 2021). Rereading the relationship between European power politics, its economic interests and the biomedicine of the past is key to imagining and planning a different future for international health coordination.

## Footnotes

1 In 1384, the sanitary council of Venice, the most important commercial port to exchange cargo with the Levant, established a rule requiring the isolation of infected ships *for a few days*. In 1465, the nearby Republic of Ragusa (today Dubrovnik) extended the isolation to forty days, giving birth to the so-called *quarantine* (McNeill 1976). Venice later retaliated by extending the administrative detention of ships from Ragusa to forty days (Marchini 2004).

2 Quarantine was also rejected as it was socially burdensome. In 1831, for example, the popular revolt which took place in the Russian Tambov administrative area against quarantine and the consequent shortages of food caused approximatively 2300 deaths and it required troops, which until 1833 militarized the area to quash the uprisings (McGrew 1960).

3 With the end of the Opium War, the Qing Empire had to concede to the establishment of so-called “treaty ports,” through which Western powers gained advantageous trade conditions (Mühlhahn 2019).
